# Bee venom-derived phospholipase A_2_ modulates microglial activity to promote antidepressant effects in a menopausal mouse model

**DOI:** 10.3389/fpsyt.2025.1694735

**Published:** 2025-11-12

**Authors:** Minsook Ye, Insop Shim

**Affiliations:** Department of Physiology, College of Medicine, Kyung Hee University, Seoul, Republic of Korea

**Keywords:** bee venom, phospholipase A2, pharmacological action, menopausal depression, antidepressant

## Abstract

**Introduction:**

Bee venom phospholipase A2 (bvPLA2) exhibits therapeutic potential in inflammatory disorders through the modulation of microglial activation, a mechanism implicated in the pathogenesis of depression. However, its effects in the context of menopausal depression remain uncharacterized. This study investigated the antidepressant effects of bvPLA2 and its underlying mechanisms in an ovariectomized (OVX) mouse model subjected to chronic restraint stress.

**Method:**

Female C57BL/6 mice were assigned to six groups: Nor (normal), Sham (the operated-only abdominal incision and non-stressed group), OVX (ovariectomized with stress), PC (positive control; estradiol-treated), bvPLA2-0.2 (0.2 mg/kg), and bvPLA2-1 (1 mg/kg). Restraint stress (2 h/day) was applied for 14 consecutive days. bvPLA2 was administered intraperitoneally, and estradiol was administered subcutaneously, once daily for two weeks. Behavioral assessments included the tail suspension test (TST), open field test (OFT), and elevated plus maze (EPM). Serum levels of corticosterone (CORT), estradiol, interleukin-1β (IL-1β), superoxide dismutase (SOD), and glutathione (GSH) were quantified via ELISA. Immunohistochemical and immunofluorescence analyses were conducted to evaluate microglial activation (CD11b), c-Fos expression, and M1/M2 polarization (CD86, CD206), with a focus on the paraventricular nucleus (PVN) of the hypothalamus.

**Results:**

bvPLA2 significantly reduced immobility time in the TST and enhanced exploratory behavior in the OFT and EPM relative to the OVX group. Treatment also lowered serum CORT and IL-1β levels and increased estradiol, SOD, and GSH concentrations, with more pronounced effects at 1 mg/kg. Furthermore, bvPLA2 attenuated microglial M1 polarization and promoted M2 polarization, suggesting suppression of neuroinflammatory responses.

**Discussion:**

These results indicate that bvPLA2 exerts antidepressant-like effects in OVX-induced menopausal depression, potentially through the modulation of neuroinflammation and oxidative stress pathways.

## 1 Introduction

Menopause is closely associated with affective disorders, as the decline in ovarian function and subsequent estrogen deficiency contribute to both central and peripheral physiological changes. This hormonal alteration induces both central and peripheral physiological changes, contributing to the development of mood-related disturbances (1, 2). The ovariectomized (OVX) rodent model is widely used to investigate menopause-related pathophysiology, as it effectively replicates postmenopausal hormonal alterations (3). Following ovariectomy, rodents exhibit increased susceptibility to anxiety- and depression-like behaviors (4). OVX rodents frequently exhibit increased vulnerability to anxiety- and depression-like behaviors, which are often exacerbated by external stressors (5). However, the precise mechanisms underlying these behavioral and neurobiological changes remain largely unknown.

The hypothalamic–pituitary–adrenal (HPA) axis plays a pivotal role in orchestrating the stress response (6). Specifically, corticotropin-releasing factor (CRF) secreted from the parvocellular division of the paraventricular nucleus (PVN) of the hypothalamus stimulates adrenocorticotropic hormone (ACTH) release from the anterior pituitary, which subsequently promotes glucocorticoid (corticosterone, CORT) secretion from the adrenal cortex in rodents (7). Dysregulation of this axis is a hallmark of stress-related mood disorders and has been consistently observed in animal models of depression (8). The relationship between estrogen deficiency, HPA axis hyperactivation, and depressive-like behavior remains an area of ongoing investigation.

Microglia, the resident immune cells of the central nervous system (CNS), play essential roles in neuroimmune surveillance, synaptic remodeling, and homeostasis. In pathological conditions, activated microglia contribute to neuroinflammation through the production of pro-inflammatory cytokines and oxidative mediators (9). Accumulating evidence indicates that microglial activation is a central feature of major depressive disorder and is often associated with behavioral impairments (9, 10). In OVX models, microglial activation is similarly elevated, suggesting that estrogen deficiency disrupts neuroimmune regulation (11). Furthermore, therapeutic interventions such as estrogen replacement, exercise, or pharmacological inhibition of inflammasomes have been shown to modulate microglial activation and alleviate OVX-induced depressive phenotypes (12).

Microglia exhibit distinct polarization states: the pro-inflammatory M1 phenotype, characterized by the release of cytokines such as interleukin-1β (IL-1β), and the anti-inflammatory M2 phenotype, associated with neuroprotection and tissue repair (13). An imbalance favoring M1 polarization contributes to sustained neuroinflammation and has been implicated in the development of depressive-like behaviors, whereas the promotion of M2 polarization is regarded as a promising therapeutic strategy (14, 15). Accordingly, examining microglial polarization in OVX-induced depressive models may provide valuable insight into the neuroimmune mechanisms underlying postmenopausal depression.

In addition to neuroinflammation, oxidative stress represents another critical factor in the pathophysiology of depression (16). Estrogen deficiency disrupts antioxidant defense systems, resulting in increased production of reactive oxygen species (ROS) and subsequent neuronal injury. Antioxidant markers such as superoxide dismutase (SOD) and glutathione (GSH) are frequently diminished in OVX models (17), thereby enhancing oxidative vulnerability and contributing to behavioral disturbances (18).

Phospholipase A2 (PLA2), a highly conserved enzyme and a major component of bee venom, catalyzes the hydrolysis of membrane phospholipids into free fatty acids and lysophospholipids, which are involved in various signaling pathways. Bee venom phospholipase A2 (bvPLA2) has been reported to exert immunomodulatory, anti-inflammatory, and neuroprotective effects in several models of inflammatory and neurodegenerative diseases, including Parkinson’s disease, Alzheimer’s disease, and atopic dermatitis (19–21). However, its potential to modulate microglial activation and oxidative stress within the context of menopausal depression remains unexplored.

This study aimed to evaluate the antidepressant effects of bvPLA2 in an OVX mouse model subjected to chronic restraint stress, with a focus on behavioral, endocrine, and neuroimmune alterations. Specifically, the study assessed depressive- and anxiety-like behaviors using the tail suspension test (TST), open field test (OFT), and elevated plus maze (EPM), and examined serum levels of CORT, estradiol, IL-1β, SOD, and GSH. In parallel, c-Fos expression and microglial M1/M2 polarization in the PVN were analyzed to elucidate the neurobiological mechanisms underlying the observed behavioral outcomes. Through this approach, the study aimed to clarify the immunoregulatory and antioxidative roles of bvPLA2 as a potential therapeutic agent for menopause-associated mood disorders.

## 2 Materials and methods

### 2.1 Animals and treatment

Female C57/BL6 mice, seven weeks old, were procured from Samtako (Osan, Korea) and maintained under controlled conditions (temperature: 22°C–24°C, 12 h light/dark cycles with lights on at 8:00 and off at 20:00). The mice received a standard diet and water ad libitum until the time of euthanasia. All experimental procedures were approved by the Institutional Animal Care and Use Committee of Kyung Hee University (KHUAP[SE]-13-041) and followed the guidelines of the National Institutes of Health Guide for the Care and Use of Laboratory Animals (revised in 1996).

bvPLA2 was purchased from Sigma-Aldrich (Cat. No. P9279, St. Louis, MO, USA) and dissolved in sterile saline immediately before use.

**Figure 1:**
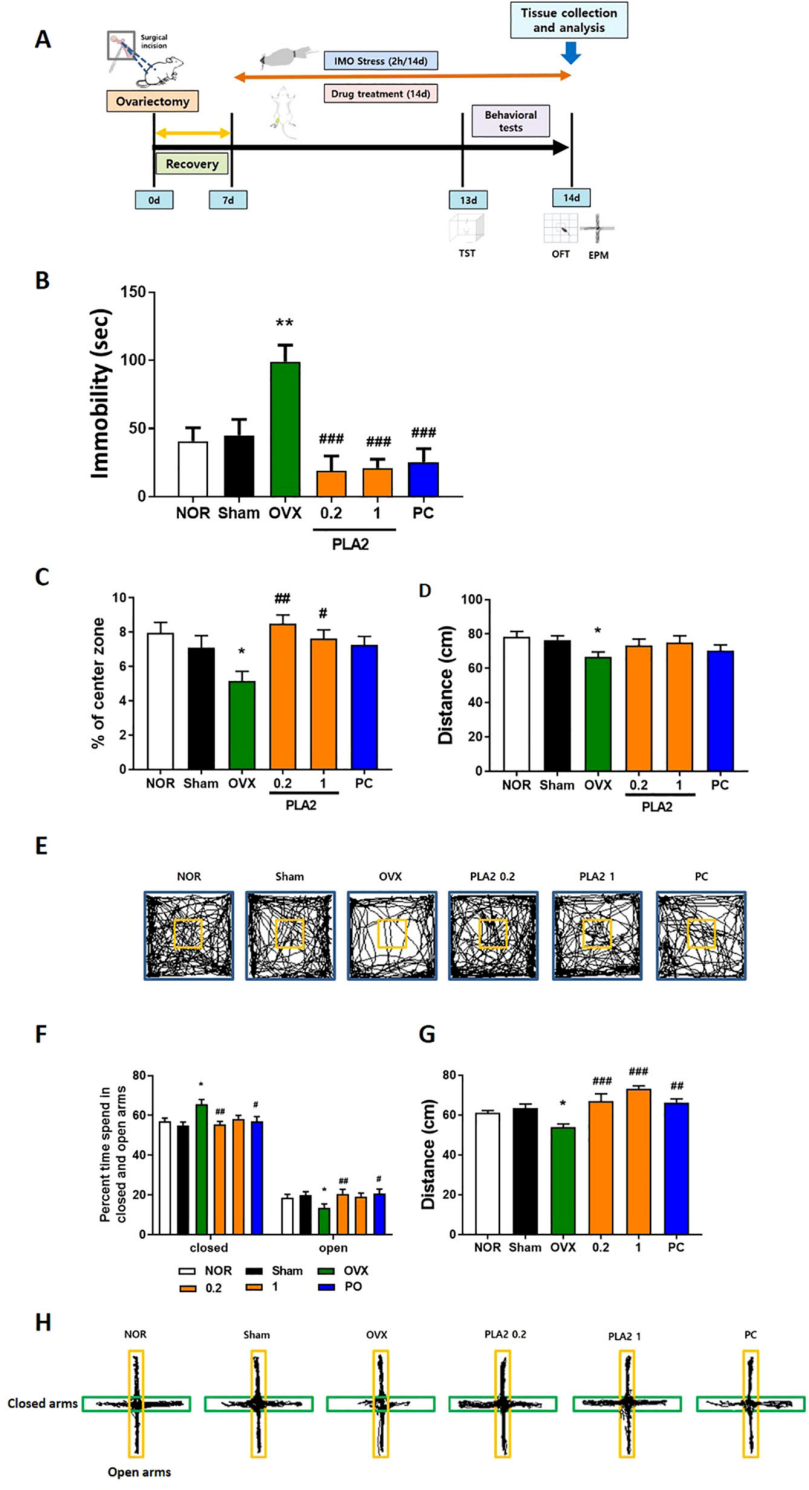
**(A)** Experimental timeline. **(B)** Effects of PLA2 on reducing immobility time in the TST in mice. **(C)** Effect of PLA2 on the percentage of time spent in the center, **(D)** total distance, and **(E)** travel pathway illustrated in the OFT test. **(F)** Effect of PLA2 on the percentage of time spent in the open arms and closed arms, **(G)** total distance, and **(H)** travel pathway illustrated in the EPM test. The data are represented as mean ± standard error of the mean (SEM) (Nor, n = 10; Sham, n = 10; OVX, n = 9; PLA2-0.2, n = 10; PLA2-1, n = 10; PC, n = 8). Statistical analysis was conducted using one-way analysis of variance, followed by Tukey’s *post hoc* test where appropriate. **p < 0.01; *p < 0.05 vs. Nor group; #p < 0.05; ##p < 0.01; ###p < 0.001 vs. OVX group.

Mice were randomly assigned to six groups: Normal control (Nor; naïve and non-stressed), Sham (the operated-only abdominal incision and non-stressed group), OVX (ovariectomized and stressed), PC (OVX, stressed, and treated with 0.2 mg/kg estradiol), PLA2-0.2 group (OVX, stressed, and treated with 0.2 mg/kg of PLA2), and PLA2–1 group (OVX, stressed, and treated with 1 mg/kg of PLA2). OVX was performed under anesthesia with pentobarbital sodium (50 mg/kg, i.p. Hanlim Pharmaceutical, Seoul, Korea). Bilateral ovaries were excised through small dorsal incisions, and the wounds were sutured. Animals were allowed a 7-day postoperative recovery period before the initiation of experiments. Immobilization stress was induced for 2 h daily (13:00–15:00) for two consecutive weeks using a disposable transparent plastic restraint cone that allowed air circulation through small openings at the tip. The Sham and OVX groups received sterile saline via intraperitoneal administration, while the other groups were administered the corresponding extract at appropriate doses once daily for two weeks. Drug administration commenced 30 min before the initiation of immobilization stress. All animals were randomly allocated to the experimental groups using a random number generator. Behavioral and biochemical evaluations were conducted by investigators blinded to the treatment conditions to minimize potential bias. To ensure experimental consistency, most procedures were performed by a single trained investigator under standardized conditions for animal handling, behavioral testing, and data collection, thereby ensuring reproducibility. The experimental schedule is illustrated in **Figure 1A**.

### 2.2 Behavioral tests

To investigate the antidepressant-like and anxiolytic mechanisms of bvPLA2, mice were subjected to the tail suspension test (TST), open field test (OFT), and elevated plus maze (EPM). All behavioral assessments were performed by an experimenter blinded to the treatment conditions, following the standardized testing sequence.

The TST, a validated method for assessing depression-like behavior, was performed after 13 days of immobilization stress and treatment. Each mouse was placed in an individual transparent acrylic chamber (20 × 20 × 30 cm) equipped with a horizontal metal bar at the top for tail suspension. The chamber was open to ensure adequate air circulation and to prevent visual contact between animals. The total duration of immobility was recorded for 5 min.

The OFT, which evaluates anxiety levels in animals by exposing them to a new environment, was conducted one day after the TST. The OFT was performed in a white acrylic box (30 cm × 30 cm × 40 cm), and the distance traveled in a central zone (10 cm × 10 cm) and the number of entries into this zone were recorded for 10 min using the SMART video tracking system (Panlab, Harvard Apparatus, Barcelona, Spain).

The EPM test, assessing anxiety-like behaviors, was conducted 2 h after the OFT. The maze apparatus consisted of a cross-shaped Plexiglas platform with two reverse open arms (50 cm × 10 cm), and two reverse closed arms (50 cm × 10 cm) connected by a central platform (10 cm × 10 cm). Behavioral parameters, including the number of entries and time spent in open and closed arms, were recorded over a 5-min test period using the SMART tracking software.

### 2.3 Measurement of corticosterone, interleukin-1 beta, estradiol, SOD, and GSH concentration

After behavioral testing, mice were anesthetized, and blood samples were collected by intracardiac puncture between 2:00 and 4:00 p.m. to minimize the effects of corticosterone diurnal variation. The samples were allowed to clot at room temperature for 30 minutes and centrifuged at 3,000 rpm for 15 minutes at 4°C to obtain serum. The serum was stored at −20°C until analysis. Serum concentrations of CORT (Enzo Life Sciences, New York, USA, Cat. No. ADI-900-097), IL-1β (Abcam, MA, USA, Cat. No. ab100705), estradiol (R&D Systems, MN, USA, Cat. No. NBP3-23559), SOD (Abcam, MA, USA, Cat. No. ab285309), and GSH (Abcam, MA, USA, Cat. No. ab239727) were measured using ELISA kits in accordance with the manufacturers’ protocols. Standard curves were prepared for each analyte, and all samples and standards were analyzed in duplicate. Optical density was determined at 450 nm using a microplate reader (Bio-Rad, CA, USA).

### 2.4 Immunohistochemistry, immunofluorescence staining and quantification for c-Fos expression, microglial inactivation, and microglial polarization

To investigate the impact of PLA2 on depression and neuroinflammation, immunohistochemical and immunofluorescence analyses were performed to assess microglial activation states, polarization, and c-Fos expression. For c-Fos immunostaining, sections underwent antigen retrieval using a 3% H2O2 buffer and PBS wash. Subsequently, sections were blocked with 1.5% bovine serum albumin and 0.2% Triton X-100 in PBS for 1 h at room temperature. Following this, sections were incubated with rabbit anti-c-Fos antibody (Santa Cruz Biotechnology, 1:1000, Cat. No. sc-52) overnight, followed by biotinylated goat anti-rabbit IgG antibody (Vector, 1:500, Cat. No. PK-6101) for 2 h at room temperature. Visualization was achieved using the Vectastain Elite ABC Kit (Vector Laboratories, Burlingame, USA) and diaminobenzidine (DAB) substrate with nickel enhancement (Vector Laboratories, Burlingame, USA). The stained tissues were examined under a bright-field microscope at regular intervals to evaluate staining quality and cellular morphology.

For double-label immunofluorescence analysis, 30-μm-thick coronal sections underwent antigen retrieval using a 3% H2O2 buffer and PBS wash. Subsequently, sections were blocked with 1.5% bovine serum albumin and 0.2% Triton X-100 in PBS for 1 h at room temperature. Sections were then incubated with rat anti-CD11b (Wako, 1:200), rabbit anti-CD86 (1:200), mouse anti-CD206 (1:200), and goat anti-c-Fos (1:500) for 12 h. Following this, sections were incubated with Alexa Fluor 594 goat anti-rat (1:200; Vector Laboratories, Burlingame, CA, USA), Alexa Fluor 488 chicken anti-rabbit (1:200; Vector Laboratories, Burlingame, CA, USA), or Alexa Fluor 594 chicken anti-mouse (1:200; Vector Laboratories, Burlingame, CA, USA) for 2 h in the dark. Fluoroshield Mounting Medium containing DAPI (Abcam). Fluorescent images were captured using an inverted fluorescence microscope (Nikon Instruments Inc., Tokyo, Japan), and quantitative analysis was conducted with Olympus imaging software to determine the mean fluorescence intensity across each visual field. Fluorescence density was calculated from eight consecutive Z-stack optical sections per sample to ensure representative quantification.

### 2.5 Statistical analysis

Statistical analyses were conducted using IBM SPSS 23.0, and the results are expressed as mean ± standard error of the mean. One-way analysis of variance, followed by Tukey’s test, was employed for statistical comparisons of behavioral and immunological outcomes. A significance threshold of p ≤ 0.05 was applied to determine statistical significance.

## 3 Results

### 3.1 Antidepressant-like effect of PLA2

In **Figure 1B**, the impact of PLA2 on the duration of immobility during the TST is depicted. Both the NOR and Sham groups exhibited significantly shorter immobility durations compared with the OVX group (p < 0.01). Treatment with PLA2 (0.2 and 1 mg/kg) and PC significantly reduced immobility times compared with the OVX group [F(5, 51)=8.595, p < 0.001].

**Figure 1C** presents the effects of PLA2 on the time spent in the center zone during the OFT [F(5, 51)=3.939]. The NOR and Sham groups spent significantly more time in the center than the OVX group (p < 0.05). Mice treated with PLA2 (0.2 and 1 mg/kg) also showed a significant increase in center time compared with the OVX group (p < 0.01 and p < 0.05, respectively). In **Figure 1D**, the total distance traveled during the OFT is presented [F(5, 51)=1.717, p=0.147]. The OVX group exhibited a significant reduction in locomotor activity compared with the NOR group (p < 0.05). PLA2 (0.2 and 1 mg/kg) and PC treatment partially restored locomotor activity. **Figure 1E** shows representative movement tracks of mice in the OFT.

In **Figure 1F**, the effects of PLA2 on time spent in the open arms [F(5, 51)=3.968] and closed arms [F(5, 51)=1.701, p=0.1498] of the EPM are illustrated. The Nor and Sham groups spent significantly more time in the open arms than the OVX group (p < 0.05). The PC and PLA2 (0.2 mg/kg) groups also exhibited significantly longer durations in the open arms compared with the OVX group (p < 0.01 and p < 0.05, respectively).

**Figure 1G** shows the total distance traveled in the EPM [F(5, 51)=9.370]. The OVX group demonstrated decreased locomotor activity relative to the Nor group (p < 0.05), whereas PLA2 (0.2 and 1 mg/kg) and PC treatments significantly restored locomotion (p < 0.01 and p < 0.001, respectively).

**Figure 1H** shows representative tracking patterns in the EPM. The OVX group exhibited reduced entries into the open arms, whereas PLA2- and PC-treated groups showed increased exploration of the open arms, indicating decreased anxiety-like behavior.

### 3.2 Effect of PLA2 on immobilization stress-induced change in the serum concentration of CORT, estradiol, IL-1β, SOD, and GSH

**Figure 2:**
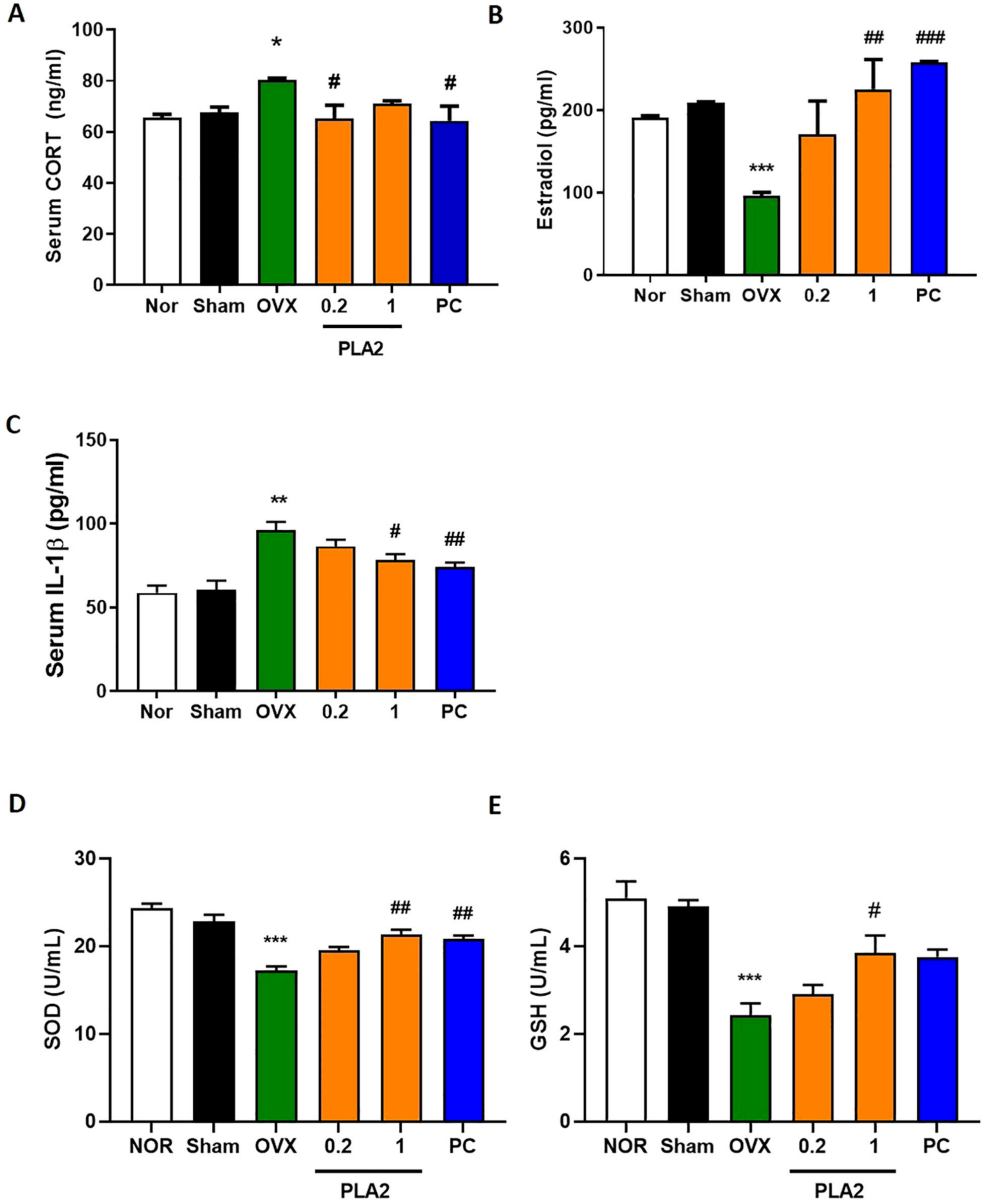
Effect of bvPLA2 on restraint stress–induced changes in serum biomarkers. **(A)** CORT (Nor, n = 8; Sham, n = 8; OVX, n = 8; PLA2-0.2, n = 8; PLA2-1, n = 8; PC, n = 8). **(B, C)** E2 and IL-1β (Nor, n = 4; Sham, n = 4; OVX, n = 4; PLA2-0.2, n = 4; PLA2-1, n = 4; PC, n = 4). **(D, E)** SOD and GSH (Nor, n = 3; Sham, n = 3; OVX, n = 3; PLA2-0.2, n = 3; PLA2-1, n = 3; PC, n = 3). Data are expressed as mean ± SEM. Statistical analysis was performed using one-way ANOVA followed by Tukey’s *post hoc* test. *p < 0.05, **p < 0.01, ***p < 0.001 vs. Nor group; **#**p < 0.05, **##**p < 0.01, **###**p < 0.001 vs. OVX group.

As shown in **Figure 2A**, serum CORT levels were significantly increased in the OVX group compared with the Sham group, indicating HPA axis hyperactivation following ovariectomy and stress exposure. Treatment with bvPLA2 (0.2 mg/kg) and estradiol significantly reduced CORT levels compared with the OVX group [F(5,42)=3.207, p < 0.05].

As presented in **Figure 2B**, serum estradiol levels were markedly decreased in OVX mice relative to the Nor and Sham groups. Treatment with bvPLA2 (1 mg/kg) and estradiol significantly restored estradiol concentrations compared with the OVX group [F(5,18)=6.158, p < 0.01 and p < 0.001, respectively].

Regarding IL-1β levels (**Figure 2C**), the OVX group exhibited a significant increase compared with controls. Both the bvPLA2 (1 mg/kg) and estradiol-treated groups showed significantly lower IL-1β levels than the OVX group [F(5,18)=11.68, p < 0.05 and p < 0.01, respectively], indicating suppression of systemic inflammation.

Serum antioxidant enzyme activity also reflected the oxidative imbalance associated with estrogen deficiency. As shown in **Figures 2D, E**, SOD [F(5,12)=20.14] and GSH [F(5,12)=14.08] levels were significantly reduced in the OVX group compared with the Sham group (p < 0.001). Treatment with bvPLA2 (1 mg/kg) significantly increased SOD and GSH levels compared with the OVX group (p < 0.05 and p < 0.01, respectively). Although estradiol treatment also increased GSH levels, the change did not reach statistical significance (p=0.0512).

Collectively, these results indicate that bvPLA2 treatment attenuates HPA axis over-activation, reduces inflammatory cytokine expression, and restores antioxidant defense capacity in OVX mice subjected to chronic stress.

### 3.3 Effects of PLA2 on c-Fos expression in the PVN

**Figure 3:**
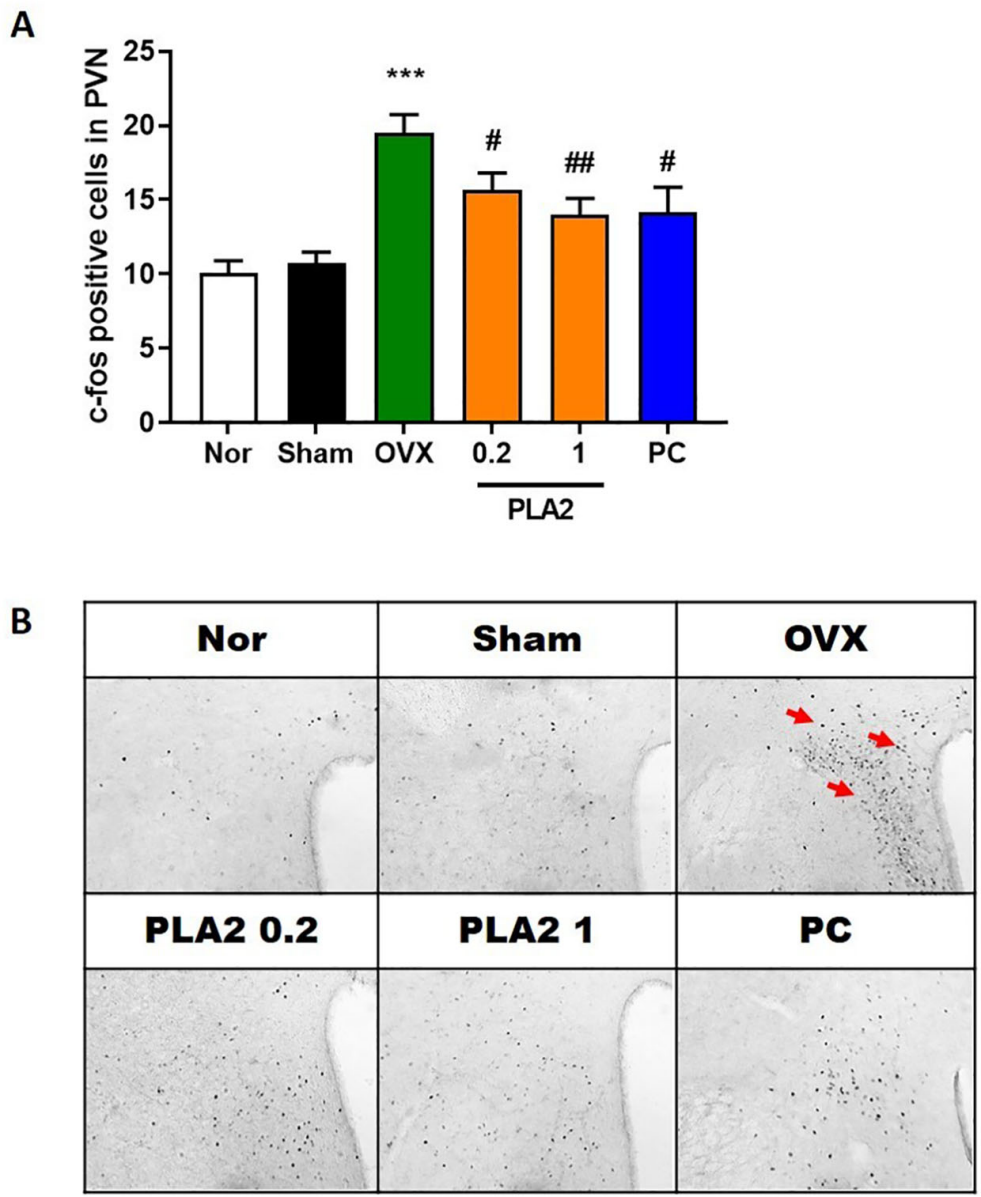
Effect of PLA2 on the expression of c-Fos–positive cells in the PVN region. **(A)** Results of c-Fos immunoreactivity were analyzed by one-way ANOVA among groups. **(B)** Representative images of c-Fos–positive cells in the PVN region. Each value represents mean ± SEM) (Nor, n = 10; Sham, n = 7; OVX, n = 9; PLA2-0.2, n = 9; PLA2-1, n = 9; PC, n = 7). ***p < 0.001 vs. Nor group; #p < 0.05; ##p < 0.01 vs. OVX group.

As shown in **Figure 3**, c-Fos expression in the PVN of the hypothalamus significantly differed among groups [F(5,43) = 9.877]. OVX mice exhibited a marked increase in the number of c-Fos–immunoreactive neurons compared with the Sham group, indicating neuronal hyperactivation induced by estrogen deficiency and chronic stress. Treatment with bvPLA2 significantly reduced c-Fos expression in the PVN compared with the OVX group (p < 0.05 and p < 0.01 for 0.2 and 1 mg/kg, respectively). Similarly, estradiol treatment also decreased c-Fos–positive cell counts (p < 0.05). These findings suggest that bvPLA2 attenuates stress-induced neuronal activation in the hypothalamus.

### 3.4 Effect of PLA2 on microglial inactivation in the brain

**Figure 4:**
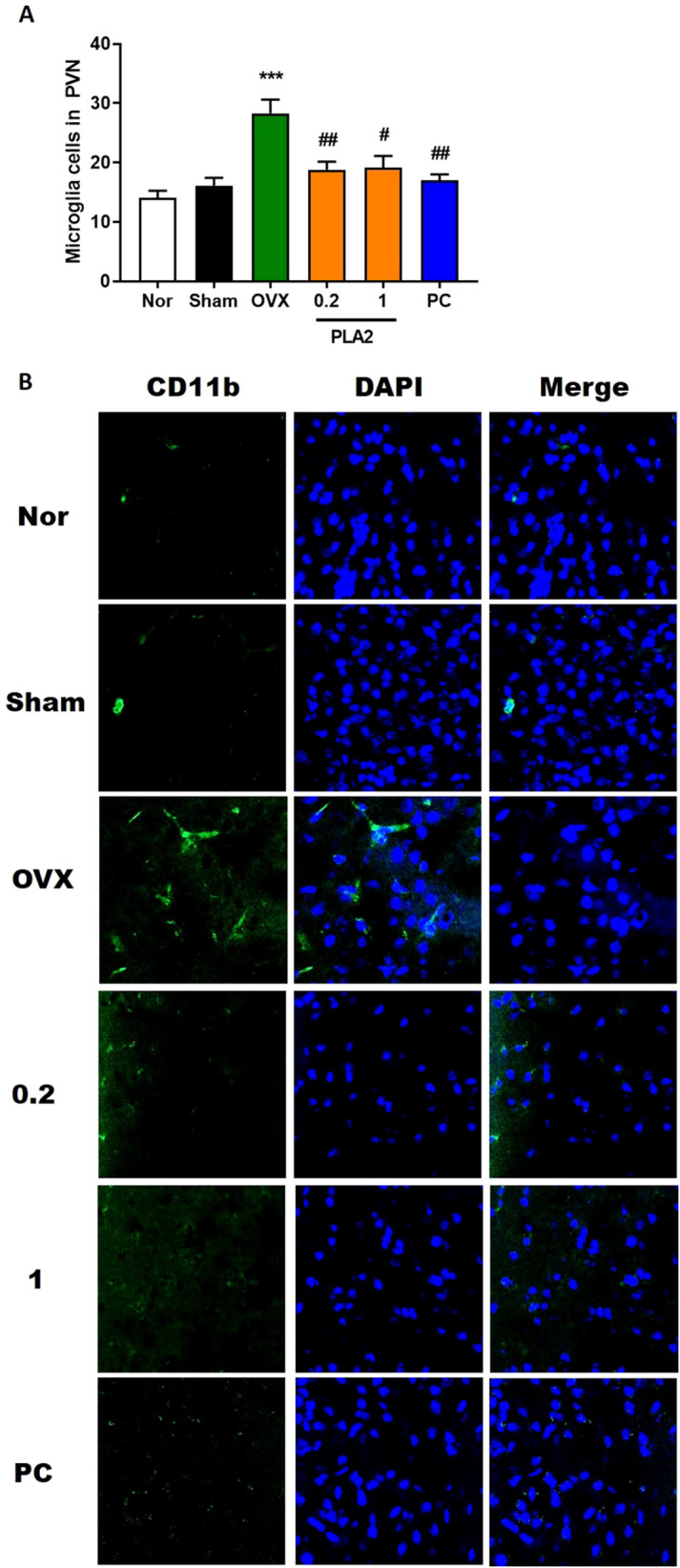
Treatment with PLA2 induces microglial deactivation in the PVN region. **(A)** Results of microglial activation were analyzed by one-way ANOVA among groups. **(B)** Sections were processed for CD11b immunofluorescence. Confocal microscopy revealed microglial fluorescence (green) in the PVN region. Nuclei were counterstained with DAPI (blue). Each value represents mean ± SEM) (Nor, n = 6; Sham, n = 6; OVX, n = 6; PLA2-0.2, n = 6; PLA2-1, n = 6; PC, n = 6). ***p < 0.001 vs. Nor group; #p < 0.05; ##p < 0.01 vs. OVX group.

As shown in **Figure 4**, the number and morphology of CD11b^+^ microglia/macrophages in the PVN differed significantly among groups [F(5,30) = 9.816]. In the Nor group, only a few CD11b^+^ microglia/macrophages with small cell bodies and thin processes were observed, indicating a resting state. In contrast, the OVX group displayed a marked increase in CD11b^+^ cells with enlarged cell bodies and thickened processes, consistent with activated microglia morphology. Treatment with bvPLA2 significantly reduced the number of CD11b^+^ microglia/macrophages in the PVN compared with the OVX group (p < 0.05 and p < 0.01 for 1 and 0.2 mg/kg, respectively), and a similar reduction was observed in the estradiol-treated group (p < 0.01). These results indicate that bvPLA2 attenuates OVX- and stress-induced microglial activation in the hypothalamus.

### 3.5 Effect of PLA2 on microglial polarization

**Figure 5:**
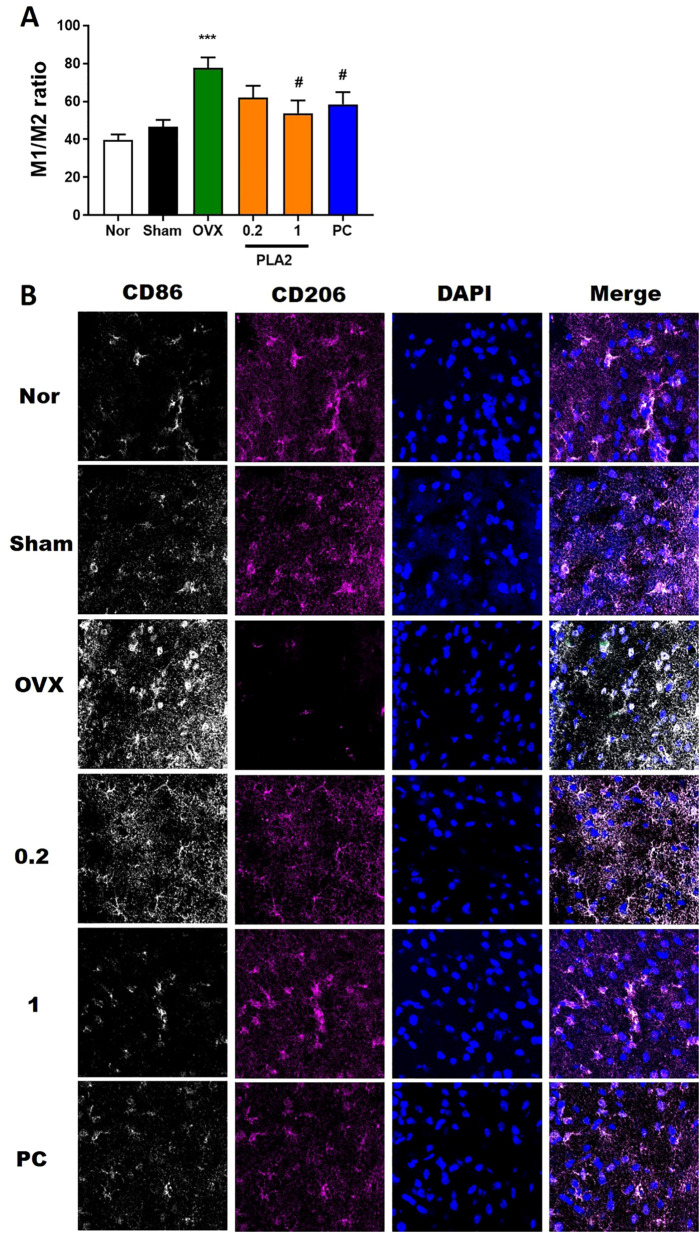
PLA2 changes the M1/M2 ratio in the PVN region. **(A)** Results of microglial activation were analyzed by one-way ANOVA among groups. **(B)** Confocal microscopic images show CD86^+^ M1 macrophage fluorescence (white) and CD206^+^ M2 macrophage fluorescence (magenta) in the PVN region. Nuclei were counterstained with DAPI (blue). Each value represents mean ± SEM (Nor, n = 6; Sham, n = 6; OVX, n = 6; PLA2-0.2, n = 6; PLA2-1, n = 6; PC, n = 6). ***p < 0.001 vs. Nor group; #p < 0.05 vs. OVX group.

While M1 microglia produce toxic substances to neurons, such as pro-inflammatory cytokines and reactive oxygen species, M2 microglia produce anti-inflammatory and tissue repair factors to promote survival and repair. We tested the effects of PLA2 on the M1/M2 microglial phenotypes using immunofluorescence double-labeling and confocal microscopy. Specifically, we measured the distribution and magnitude of macrophages expressing M1 (CD86) and M2 (CD206) phenotypic markers (**Figure 5**). The M1:M2 ratio decreased markedly upon PLA2 and PC treatment, mostly because of the increased labeling for M2 phenotypic markers [F(5,30) = 5.960, p<0.05, **Figure 5**].

## 4 Discussion

The present study showed that bvPLA2 alleviated depression-like and anxiety-like behaviors in OVX mice subjected to chronic restraint stress. bvPLA2 treatment reduced immobility time in the TST and enhanced exploratory behavior in the OFT and EPM, indicating both antidepressant- and anxiolytic-like effects. These behavioral improvements were accompanied by restoration of HPA axis function, suppression of neuroinflammation, and recovery of redox balance.

The TST is widely used to evaluate antidepressant activity, where reduced immobility indicates lower behavioral despair (22). In this study, β-estradiol, used as a positive control, significantly decreased immobility time, consistent with previous results (23). Similarly, bvPLA2-treated mice showed a marked reduction in immobility compared with the OVX group, suggesting that bvPLA2 exerts a comparable antidepressant-like effect. To minimize potential confounding effects between behavioral tests, the EPM was conducted 2 hours after the OFT. This interval was sufficient for mice to recover from handling and environmental exposure, as reported in previous studies, and thus was not expected to influence anxiety-related behaviors measured in the EPM.

The OFT and EPM were utilized to evaluate anxiety-like behavior and locomotor activity (24). OVX and stressed mice spent less time in the center zone of the OFT and in the open arms of the EPM, reflecting increased anxiety. bvPLA2 administration reversed these behaviors without affecting total locomotion, indicating a genuine anxiolytic-like effect rather than hyperactivity.

Menopause is associated with dysregulation of the HPA axis, a core system involved in stress response regulation (25). Estrogen deficiency enhances glucocorticoid secretion, particularly corticosterone in rodents, and promotes inflammatory cytokine release (26). In this study, OVX mice exhibited elevated serum CORT and IL-1β levels, consistent with neuroendocrine and inflammatory imbalance observed in depression. bvPLA2 treatment lowered both CORT and IL-1β concentrations and restored estradiol levels, suggesting that bvPLA2 contributes to normalization of HPA axis activity and attenuation of systemic inflammation in postmenopausal depression (27) (28).

Although bvPLA2 treatment increased serum estradiol levels in OVX mice, this elevation is unlikely to fully account for the observed behavioral improvements. Previous studies have reported that bvPLA2 exerts immunomodulatory and neuroprotective effects through acetylcholine receptor–mediated and regulatory T cell–dependent pathways, independent of estrogen signaling (21, 29, 30). Therefore, while partial restoration of estradiol may have contributed to the behavioral outcomes, the present findings indicate that bvPLA2 primarily exerts its effects through direct neuroimmune regulatory mechanisms.

c-Fos expression in the paraventricular nucleus (PVN) of the hypothalamus is commonly regarded as an indicator of neuronal and cellular activation associated with stress responsivity. OVX and chronic stress also increased c-Fos expression in the PVN, indicating neuronal hyperactivation, while bvPLA2 treatment significantly reduced c-Fos expression, implying modulation of PVN activity and improved stress regulation.

Neuroinflammation plays a central role in the development of depression. In OVX mice, microglial activation shifted toward the pro-inflammatory M1 phenotype, as evidenced by increased CD86 and reduced CD206 expression. bvPLA2 reversed this pattern, promoting M2 polarization and reducing overall microglial reactivity. These findings indicate that bvPLA2’s antidepressant effects may be mediated through regulation of microglial phenotype, consistent with prior studies showing the therapeutic potential of microglial modulation in mood disorders (12).

However, although the present data indicate a clear association between microglial polarization and behavioral improvement, they do not establish a direct causal relationship. Additional studies employing microglial inhibitors or conditional knockout models are required to determine whether the behavioral effects of bvPLA2 are directly mediated through microglial modulation or occur via parallel mechanisms.

Estrogen deficiency also impairs antioxidant defense systems, increasing oxidative stress and neuronal vulnerability. Reduced SOD and GSH levels in OVX mice confirmed oxidative imbalance, whereas bvPLA2 restored both antioxidants, suggesting enhanced redox stability and protection against oxidative injury. Thus, bvPLA2 exerts both anti-inflammatory and antioxidant neuroprotective effects.

Mechanistically, PLA2 hydrolyzes membrane phospholipids to produce bioactive lipids such as arachidonic acid and lysophospholipids, which can modulate immune responses (31). Previous work has shown that bvPLA2 suppresses neuroinflammatory signaling and protects neurons in Parkinson’s disease models (29). The present findings extend this evidence by showing that bvPLA2 mitigates neuroinflammation and oxidative stress in a menopausal depression model.

In conclusion, bvPLA2 exerts antidepressant-like effects in OVX mice by improving mood-related behaviors, normalizing HPA-axis activity, reducing inflammation, and enhancing antioxidant capacity. These findings support bvPLA2 as a potential therapeutic candidate for postmenopausal depression, although further studies are needed to clarify the molecular pathways involved, such as NF-κB and PPARγ signaling.

## Data Availability

The original contributions presented in the study are included in the article/supplementary material. Further inquiries can be directed to the corresponding author.
